# Detection of solar QBO-like signals in earth’s magnetic field from multi-GOES mission data

**DOI:** 10.1038/s41598-023-46902-6

**Published:** 2023-11-09

**Authors:** Fadil Inceoglu, Paul T. M. Loto’aniu

**Affiliations:** 1 Cooperative Institute for Research in Environmental Sciences, University of Colorado Boulder, Boulder, CO 80309 USA; 2 National Centers for Environmental Information, National Oceanic and Atmospheric Administration, Boulder, 80309 CO USA

**Keywords:** Magnetospheric physics, Solar physics

## Abstract

Through variations in its magnetic activity at different timescales, the Sun strongly influences the space weather conditions throughout the heliosphere. The most known solar activity variation is the Schwabe Cycle, also known as the Sunspot Cycle (SCs), period of which ranges from 9 to 13 years. The Sun also shows shorter quasi-periodic variations, such as the quasi-biennial oscillations (QBOs), superposed on the SCs. The QBOs are thought to be a global phenomena extending from the subsurface layers of the Sun to Earth and throughout the Heliosphere with a period generally between 1.3 and 1.6 years. In this study, we, for the first time, detected signals with periods ranging from 1.3 to 1.6 years in Earth’s magnetosphere, which can be associated with the solar QBOs, using data from multiple GOES missions. The QBO-like signals detected in Earths Magnetopshere are thought to be propagated via the solar wind from the solar surface.

## Introduction

Space weather conditions throughout the heliosphere is strongly influenced by magnetic activity at the Sun that have different cyclical timescales. The most well-known magnetic activity cycle is the Schwabe Cycle^1^, which is also called the Solar Cycle (SC), the periods of which range from 9 to 13 years. In addition to the SCs, the Sun also shows shorter quasi-periodic variations, such as the 160-day Rieger-type periodicities^2^ and quasi-biennial oscillations (QBOs) with a period that ranges from 0.6 to 4 years, with most common periods found between 1.3 and 1.6 years^3^.

The QBOs are intermittent signals and they are in-phase with the SCs, attaining their maximum (minimum) amplitudes during SC maxima (minima)^3^. They are present over all solar latitudes and exhibit different behaviors in each solar hemisphere^4–8^. The QBO signals are detected in the immediate subsurface layers of the Sun, H$\alpha$ flare activity in the northern and southern solar hemispheres, high-latitude polar faculae numbers in the northern and southern solar hemispheres, CME occurrances in the north-south component of the interplanetary magnetic field, geomagnetic activity indices, solar wind speed measurements throughout the Heliosphere as well as in Galactic Cosmic Ray intensities^4,7–16^. Recently, it was shown that the QBO signals can also be detected in the differential rotation rate residuals with amplitudes increasing with increasing depth^17^. Their maximum amplitude are found to be around 55$^{\circ }$ and below **0.78**$R_{\odot }$, exhibiting a decrease with decreasing latitude^18^. Therefore, the QBOs are thought to be global phenomena extending from the subsurface layers of the Sun to Earth and throughout the Heliosphere.

The most prevailing physical explanation for the existence of the QBOs comes from instability of the magnetic Rossby waves in the tachocline^19^ and tachocline nonlinear oscillations (TNOs) where periodic energy exchange takes place among the Rossby waves, differential rotation, and the present toroidal field^20^. In addition, based on modeling results using fully nonlinear flux transport dynamos, it was proposed that the mechanism for generating QBOs occurs via the interplay between plasma flow and magnetic fields, with the turbulent ${\alpha }$-mechanism working in the lower half of the solar convection zone, extending from 0.70R$_{\odot }$ to the surface^6^. These results were supported by observational studies using differential rotation rate residuals and photospheric magnetic field measurements from the Michelson Doppler Imager on the Solar and Heliospheric Observatory and Helioseismic and Magnetic Imager on the Solar Dynamics Observatory^17,18^. The bottom of the convection zone overlaps with the region of strong radial shear. Above this region a differential rotation pattern that depends strongly on latitude takes place^21^.

The QBOs as periods of high solar activity, superposed on the SCs, will lead to increases in coronal hole (CH) formations from more emerging active regions on the photosphere^22^ as well as increase in flaring and coronal mass ejection (CME) activities^7,23^. The CHs are defined as regions with low density collisionless plasma, where open magnetic field lines extend into the interplanetary medium^24,25^. The CHs are a source region for fast solar winds^26^, which cause geoeffective space weather events^27^. A source for severe space weather activity, which could dramatically effect our space and ground-based infrastructure, are CMEs that accelerate through the fast and slow solar wind streams^28^.

Different from the past studies that considered only indirect indices that represent geomagnetic activity, in this study, for the first time, we detected signals with periods ranging from 1.3 to 1.6 years in Earth’s Magnetosphere, which can be associated with the solar QBOs. This objective was accomplished using measurements of Earth’s magnetic field from multiple Geostationary Operational Environmental Satellite (GOES) missions covering the past three SCs. Measurements of Earth’s magnetic field at geostationary orbit are useful for scientific and operational purposes as this location is effective for monitoring major magnetospheric current systems^29^.

## Results

**Figure 1:**
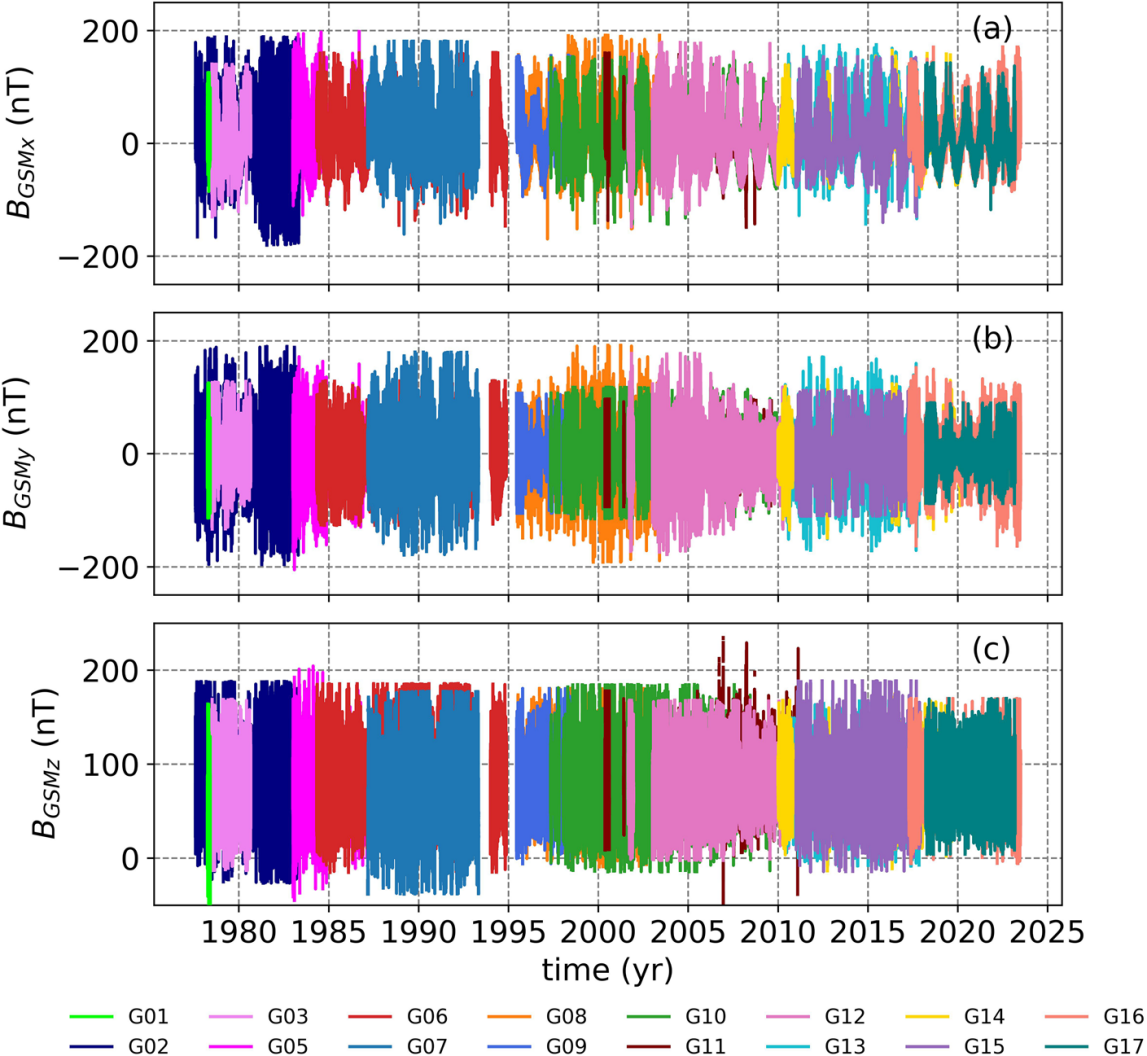
The minute-resolution magnetic field measurements from GOES-01 to GOES-17 in GSM coordinates for a time range from 1977 to 2023.

The minute-resolution magnetic field measurements from 16 GOES missions in GSM coordinates show overlapping periods where there are two or more satellites measuring Earth’s magnetic field at geostationary orbit (the left panels of Fig. 1). Even though we are using data covering from GOES-01 to GOES-17, there are two periods in 1993 and 1995 where no GOES measurements were available. For all components of the GSM frame (B$_{GSMx}$, B$_{GSMy}$, and B$_{GSMz}$), although different GOES missions show similar variations, some offsets between GOES missions can also be observed (Fig. 1).

**Figure 2:**
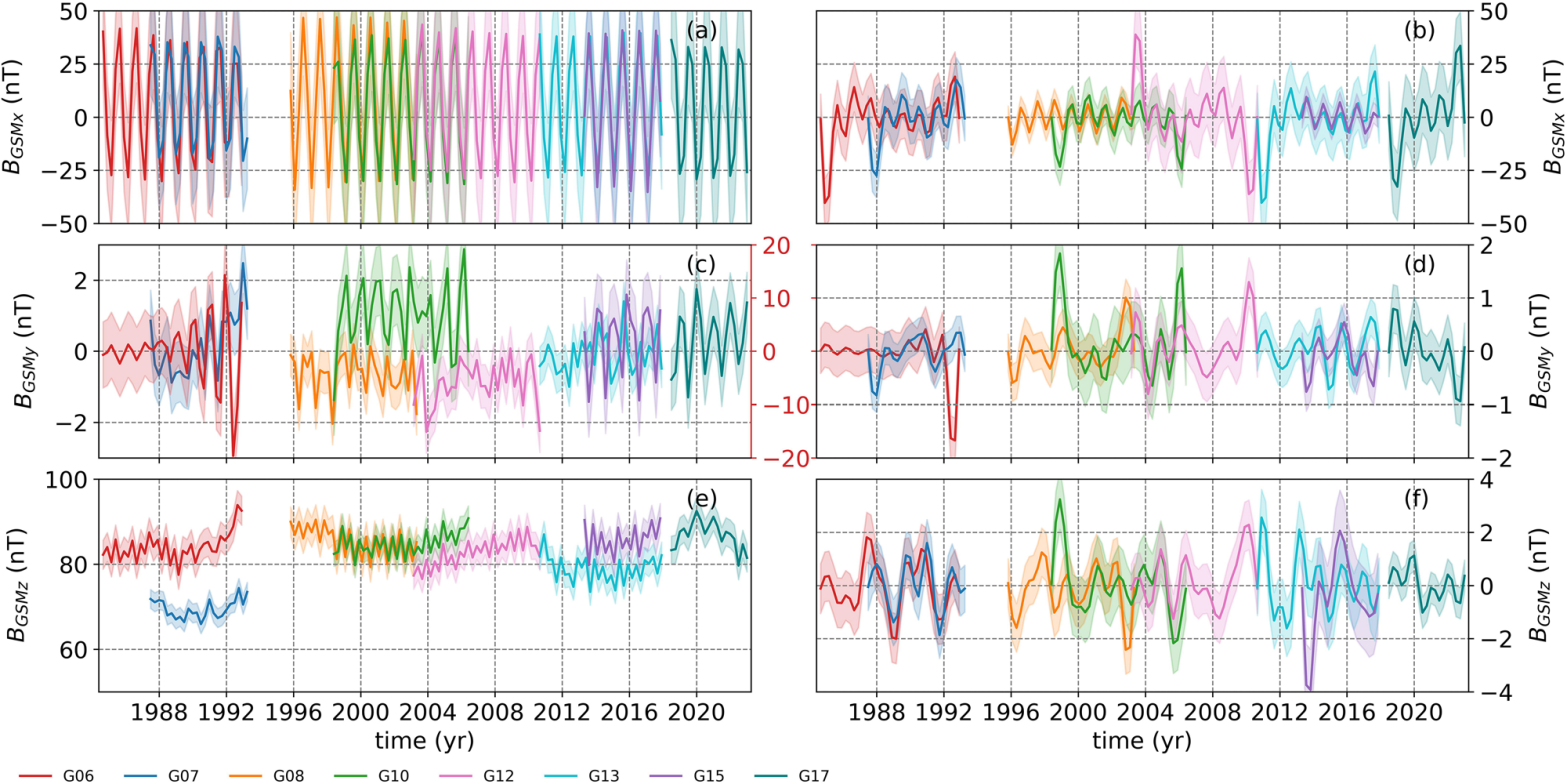
The left panels show 3-month averaged magnetic fields in GSM coordinates (**a**, **c**, and **e**, respectively). The right panels show the same, but for the bandpass-filtered data for the frequency band between (1/4.5) and (1/1.1) year$^{-1}$. The shaded areas on the left panels show 1$\sigma$ standard deviations calculated using 3-month binned data, while the shaded areas on the right panels show the 1$\sigma$ standard deviations. Note that panels (**c**) and (**d**) have a second y-axes for GOES-06, which are marked with red font.

As we are interested in the variations above 1-year, we calculated the 3-month averages of the geomagnetic field in GSM coordinates and applied our data selection criteria to data from all the GOES missions (see “Methods”). The data selection criteria resulted in magnetic field data from 8 GOES missions with durations ranging between 4.5 and 8.2 years (Fig. 2a,c,e). The sinusoidal variation in the B$_{GSMx}$ component (Fig. 2a) is due to the orbital configurations of the GOES satellites relative to the ecliptic plane. The projection of Earth’s dipole field onto the GSM x-axis is positive (negative) when the north magnetic dipole tilts towards (away) from the Sun creating the 6-month periodicity in the data. The B$_{GSMz}$ component is the closest component to the geomagnetic dipole and therefore generally has the largest average value.

Even though B$_{GSMx}$ values for the 8 GOES missions overlap, there are clear offsets both in B$_{GSMy}$ and B$_{GSMz}$ components (Fig. 2c,e). For example, in B$_{GSMy}$, GOES-10 shows an offset about 1.5 nT compared with GOES-08 and GOES-12, which show similar values. On the other hand, GOES-06 shows the largest fluctuations reaching almost 30 nT variations around 1992 (Fig. 2c). We believe GOES-06 measurements for B$_{GSMy}$ component is contaminated and might not reflect the real magnetospheric conditions. For the B$_{GSMz}$ component, the GOES-07 measurements show an offset of around 10 nT compared with the GOES-06, which is at the same level as the rest of the GOES missions under consideration (Fig. 2e). In addition to GOES-07, GOES-15 values seem to have a slight offset around 5 nT compared with GOES-13 during the same period (Fig. 2e).

Following that, to remove the effects from the orbital configurations, rotation and possible and known dominant SCs influences^3,17^ and to enhance possible QBO signatures, we applied a Butterworth bandpass filtering to the data for the frequency band between (1/4.5) and (1/1.1) year$^{-1}$ (the right panels of Fig. 2). The filtering process also helped eliminate the offsets among the data from the 8 GOES missions, except for the large amplitude dip observed in the B$_{GSMy}$ component from GOES-06 around 1992 (Fig. 2d). Another feature can be seen is the edge effects caused by the Butterworth filter, which are especially prominent in the B$_{GSMx}$ component (Fig. 2b).

**Figure 3:**
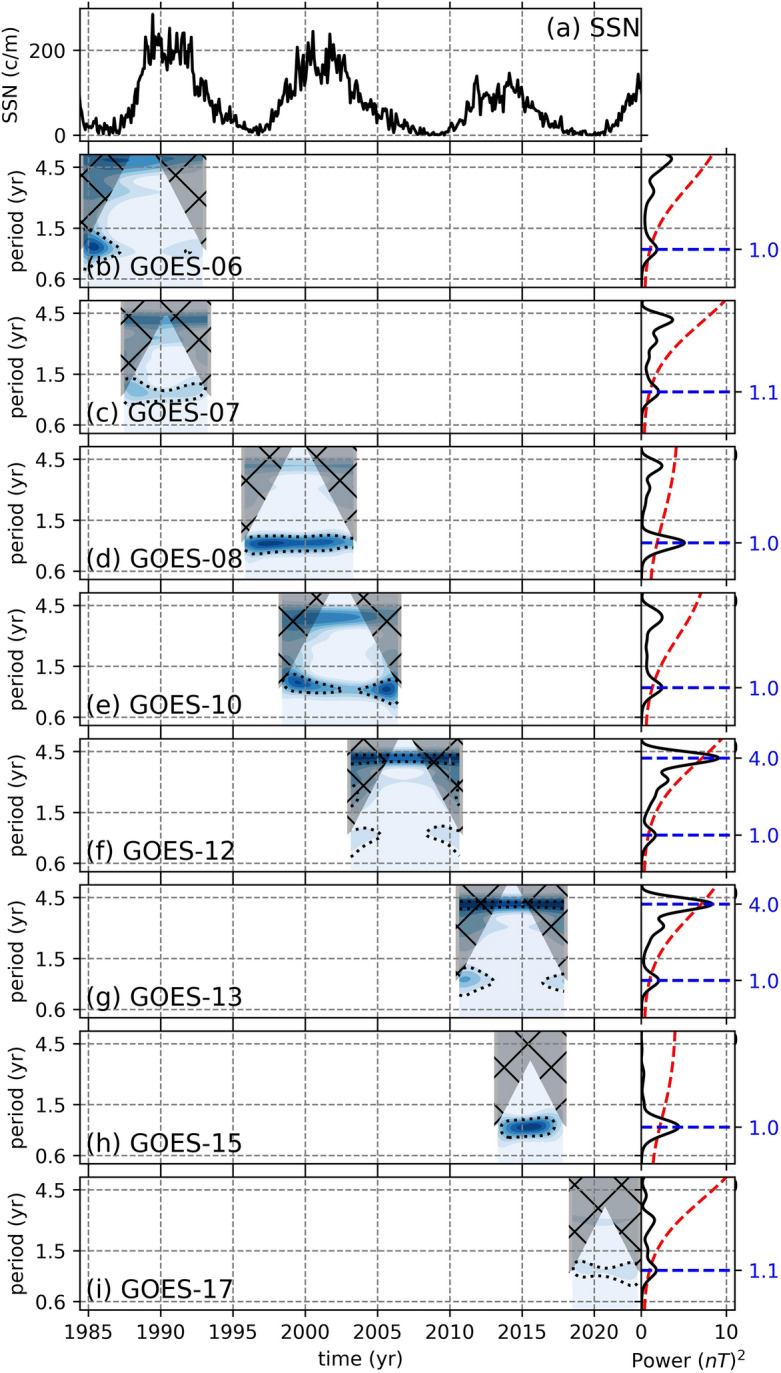
Continuous wavelet transformations of the B$_{GSMx}$ components from 8 GOES missions. The black dotted lines show statistical significance levels at $p < 0.05$. The hatched gray shaded areas show the cone of influence. On the right, attached to each CWT is their global wavelets (black solid line) with their 95% conficende spectrum (red dashed) and the statistically significant periods (blue dashed lines). The top panel shows the monthly sunspot numbers obtained from WDC-SILSO, Royal Observatory of Belgium, Brussels^30^ to recognize the phase of the SC. Note that the y-axes are in log-scale.

The periodicities in the filtered data were determined using the continuous wavelet transformation (CWT) and global wavelet (GW) spectrum methods. The resulting CWT and GW suggest that the B$_{GSMx}$ component from 8 GOES mission does not show any signals which might be associated with the solar QBOs. The signals with 1 year period present in this component is most likely related to the orbital configuration of the GOES missions (Fig. 3). Statistically significant signals with 4-year periods observed in GOES-12 and GOES-13, which are also present in GOES-06 to GOES-10 but not significant, are potentially resulting from the edge effects caused by the Butterworth filter (Figs. 3f,g).

**Figure 4:**
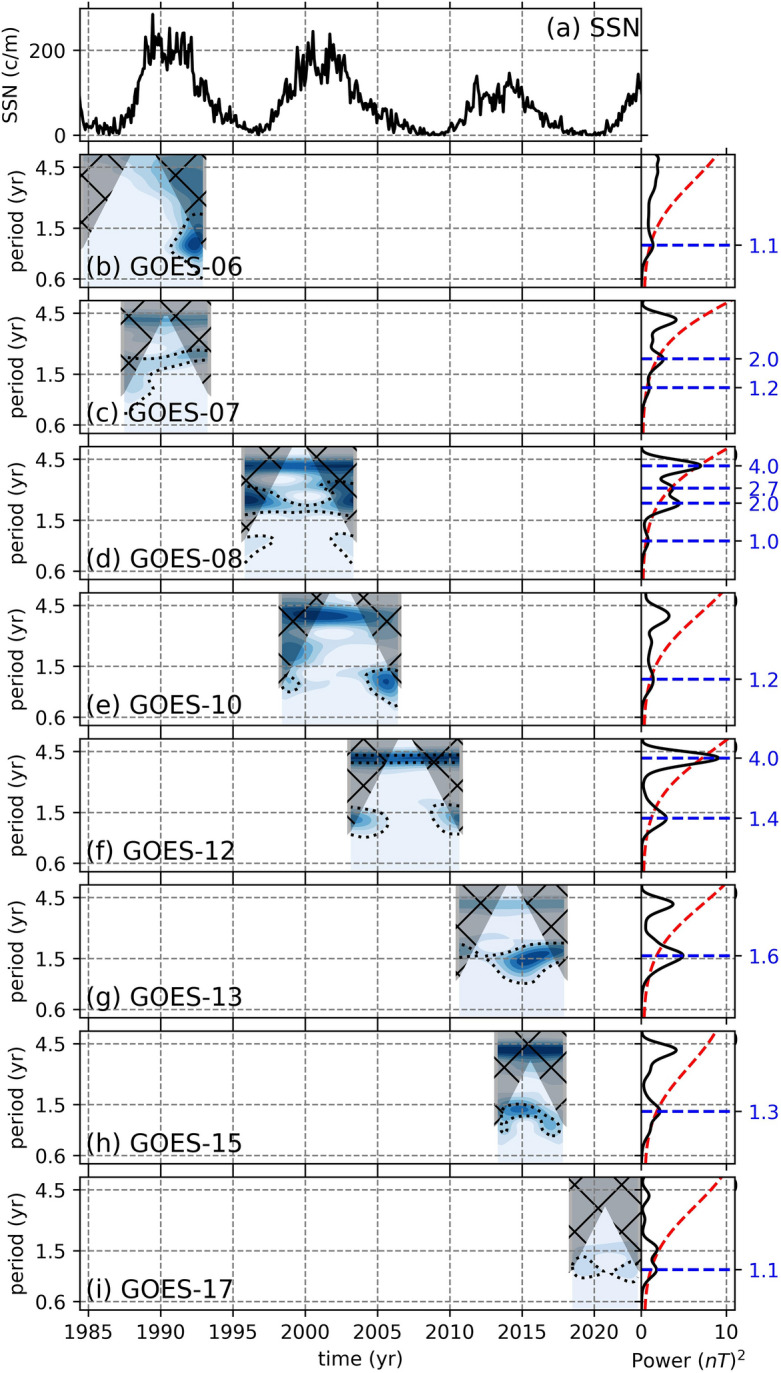
Continuous wavelet transformations of the B$_{GSMy}$ components from 8 GOES missions. The black dotted lines show statistical significance levels at $p < 0.05$. The hatched gray shaded areas show the cone of influence. On the right, attached to each CWT is their global wavelets (black solid line) with their 95% conficende spectrum (red dashed) and the statistically significant periods (blue dashed lines). The top panel shows the monthly sunspot numbers obtained from WDC-SILSO, Royal Observatory of Belgium, Brussels^30^ to recognize the phase of the SC. Note that the y-axes are in log-scale.

For the B$_{GSMy}$ component, we observe QBO-like signals with periods ranging from 1.3 to 2 years (Fig. 4). The B$_{GSMy}$ data from GOES-06, which might be contaminated (Fig. 2), exhibit a weak signal around 1.1 year after 1990, however since the data is likely contaminated and the signal is close to cone of influence, we must consider this signal as unreliable (Fig. 4b). The other GOES missions, on the other hand, show signals that might be associated with the solar QBOs. For example, GOES-07 displays a signal with 1.2 year period before 1990, which becomes longer around 2 years after the maximum of SC-22 (Fig. 4c). During SC-23 and up until the onset of the declining phase of the cycle, GOES-08 exhibits a stronger and continuous signal with 2 year period (Fig. 4d), while during the declining phase of SC-23, GOES-10 show a very weak signal with 1.2 year period (Fig. 4e) and GOES-12 show an intermittent signal with 1.4 year period toward the end of SC-23 (Fig. 4f). During SC-24, on the other hand, both GOES-13 and GOES-15 show signals with 1.6 and 1.3 year periods, respectively (Fig. 4g,h). On the onset of SC-25, GOES-17 show a very weak signal with 1.1 year period. In summary, during the maxima of each solar cycle, we observe periodicities; 2 years in GOES-07 during SC-22, 2 years in GOES-08 during SC-23, and 1.6 and 1.3 years during SC-24 in GOES-13 and GOES-15, respectively. These periodicities are consistent with solar QBOs.

**Figure 5:**
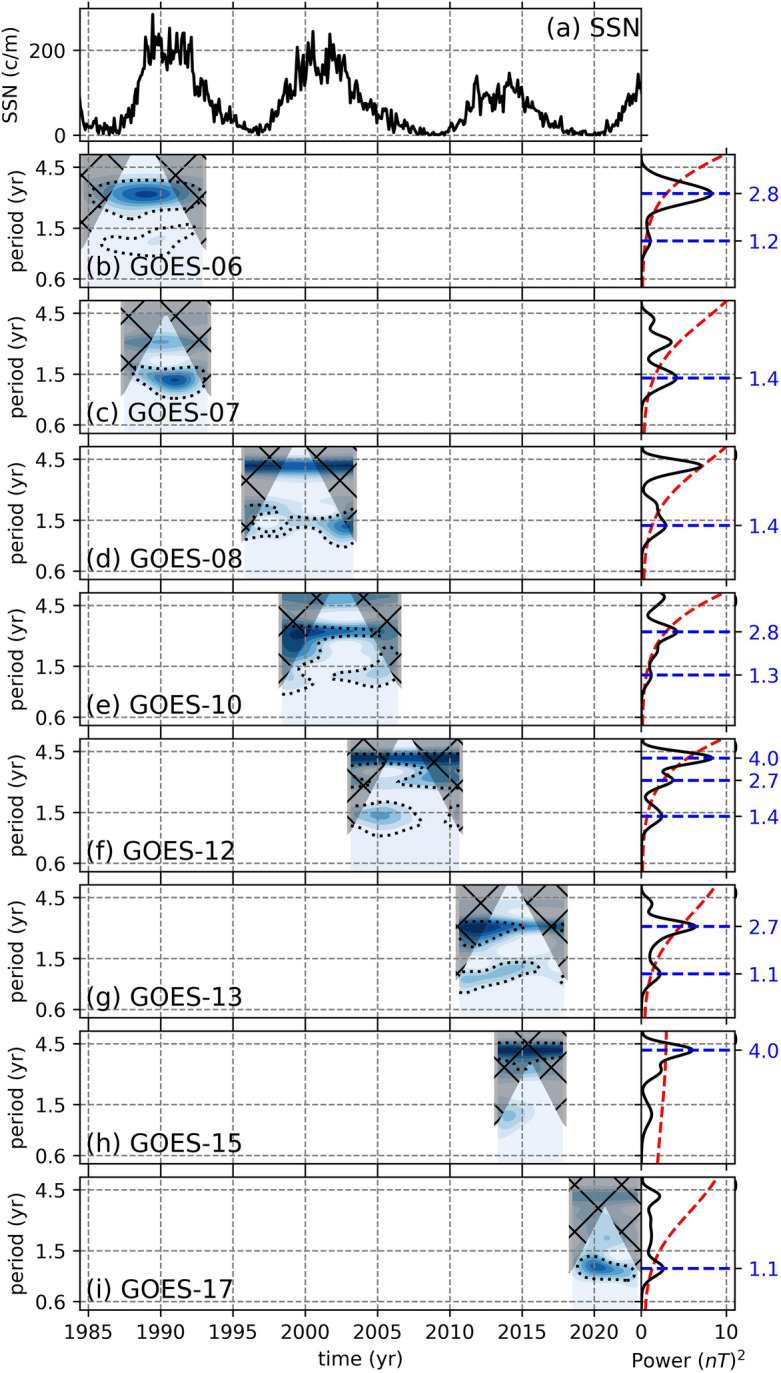
Continuous wavelet transformations of the B$_{GSMz}$ components from 8 GOES missions. The black dotted lines show statistical significance levels at $p < 0.05$. The hatched gray shaded areas show the cone of influence. On the right, attached to each CWT is their global wavelets (black solid line) with their 95% conficende spectrum (red dashed) and the statistically significant periods (blue dashed lines). The top panel shows the monthly sunspot numbers obtained from WDC-SILSO, Royal Observatory of Belgium, Brussels^30^ to recognize the phase of the SC. Note that the y-axes are in log-scale.

The B$_{GSMz}$ component also displays signals with periods around 1.4 years during the solar cycle maxima phases (Fig. 5a), which can be associated with the solar QBOs. The clearest signals can be observed in GOES-07 during SC-22, GOES-08, GOES-10, and GOES-12 through SC-23 (Fig. 5b,c,d,e,f). For SC-24, on the other hand, a signal with around 1.1 year period becomes longer about 1.5 year toward 2015 in GOES-13 (Fig. 5g), after which it vanishes. During the declining and minimum phases of SC-24, the CWT and GW of the GOES-15 data does not show any relevant periodicity, while the CWT and GW of the GOES-17 data display only a small amplitude peak with a period of 1.1 year, which might not be related to the solar QBOs (Fig. 5h,i). The peaks around 2.8 year mark in global wavelets are thought to be the second harmonics of the shorter period around 1.4 years.

**Figure 6:**
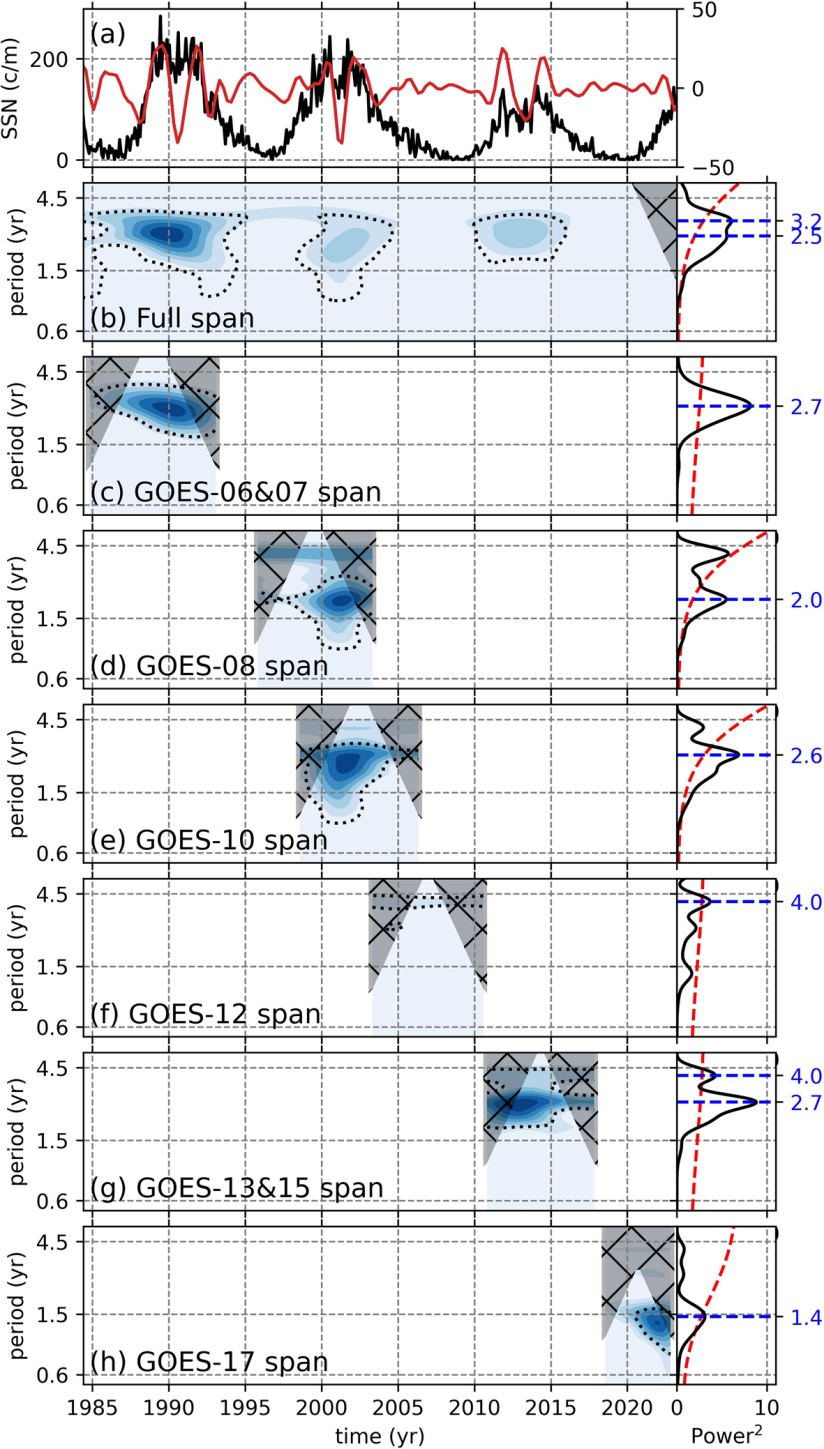
Continuous wavelet transformations of the SSNs. The black dotted lines show statistical significance levels at $p < 0.05$. The hatched gray shaded areas show the cone of influence. On the right, attached to each CWT is their global wavelets (black solid line) with their 95% conficende spectrum (red dashed) and the statistically significant periods (blue dashed lines). The top panel shows the monthly sunspot numbers obtained (black) from WDC-SILSO, Royal Observatory of Belgium, Brussels^30^, while the 3-month averaged and band-pass filtered SSNs are shown in red. The panel-(**b**) shows the CWTs for the full span of the data, while the following panels show the span of GOES-06 and-07 data (**c**), GOES-08 data (**d**), GOES-10 data (**e**), GOES-12 data (**f**), GOES-13 and-15 data (**g**), and GOES-17 data. Note that the y-axes are in log-scale.

In addition to the geomagnetic field components, we investigated the existence and behavior of the QBOs in the sunspot data to establish a connection between the solar and terrestrial magnetic fields (Fig. 6). For the full duration of the band-passed SSN data (Fig. 6a), the QBOs are present and display an intermittent behavior with stronger signals with periods 2.5–3.2 years during the solar cycle maxima, while they disappear during solar cycle minima (Fig. 6b). Following this step, we sliced the band-pass data to cover only the periods where GOES data is available. During the GOES-06 and-07 period, which overlaps with GOES-06 and GOES-07 starting slightly earlier and ending slightly later, the band-passed SSNs exhibit a strong signal with a period of 2.7 years, which becomes shorter towards the end of SC-22 (Fig. 6c). As for the GOES-08 time span, the band-passed SSNs show a strong signal with a period of 2 years, while for GOES-10 the band-passed SSNs show a QBO signal with a period of 2.6 years that is shorter on the onset of the cycle (Fig. 6d,e). During the time span of GOES-12, which also overlaps with the declining and minimum phases of SC-23, on the other hand, the band-passed SSNs only show a very weak signal with a period of around 4 years (Fig. 6f). During the time span covered by GOES-13, 15, and-17, the band-passed SSNs show QBO-like signals with periods of 4.0, 2.7, and 1.4 years (Fig. 6g,h).

## Discussion

The B$_{GSMy}$ and B$_{GSMz}$ components of the Earth’s magnetic field in GSM coordinates measured at geostationary orbit are generally more perturbed during increased solar activity with Earth’s magnetosphere reacting to variations in solar activity levels. Our results show that the B$_{GSMy}$ and B$_{GSMz}$ components of Earth’s Magnetic field display signals within the period range of the solar QBOs.

The B$_{GSMy}$ of Earth’s magnetic field, which is the azimuthal component and is positive towards dusk magnetic local time, shows signals with periods ranging from 1.3 to 2 years which can be associated with the solar QBOs, especially during solar cycle maxima. During solar cycle maxima conditions, the number of equatorial CH, their areas, and the number of CME occurrences are greater^31^. The response of B$_{GSMy}$ to QBOs can be explained by merging caused by local magnetic shear and day-side merging with dawn-dusk asymmetric reconnection^32^ under increased solar activity conditions in QBO time-scales.

The B$_{GSMz}$ component of the geomagnetic field, which is the cross product of the B$_{GSMx}$ and B$_{GSMy}$, displays signals with periods around 1.4 years during SCs-22 and 23. During SC-24, which is much weaker compared with SCs-22 and 23, the higher amplitude variation has a period of 2.7 years, which is not observed for the B$_{GSMy}$ component. This is similarly explained by the increased number of equatorial CHs with larger areas and increased number of CME occurrences during the QBOs, superposed on the SCs^31^, and hence increased QBO conditions. During northward IMF periods, $\sim$ 30% of the IMF uniformly penetrates the B$_{GSMz}$ that is observed at geostationary orbit, while for southward IMF periods the penetration is much larger across all local times because of enhanced cross-tail currents^32^.

We also considered effects arising from semi-annual variability which has been long known that the geomagnetic activity levels exhibit. Three mechanisms are attributed to the semi-annual variations, which maximize around the equinox, and they are the Axial, Equinoctial, and Russel & McPherron effect^33,34^. Hence, we should observe increased power at half a year and its harmonics. To avoid these effects, we used a lower cutoff frequency of (1/1.1) year$^{-1}$ for our Butterworth filter. In addition, the temporal resolution of the periods both in CWT and GWs for periods around 1 year (2nd harmonic of the semi-annual effects) is 0.011 year in average, and for periods around 1.5 years (3rd harmonic of the semi-annual effects) is 0.016 year in average. Therefore, we concluded that the resolution of the periods in CWT and GW is high enough to differentiate the semi-annual effect and its potential harmonics from the statistically significant signals of 1.3, 1.4, 1.6, and 2 years observed in the B$_{GSMy}$ and B$_{GSMz}$ components and they can be associated with the solar QBOs.

We also detected QBO signals in the band-passed SSN data for the full span of the study period as well as time spans where the data from the various GOES missions were available. In addition to the existence of the QBOs in the SSN data, previous studies show that the QBO signals were found in solar surface magnetic field, emergence of polar faculae, H$\alpha$ flaring activity, and CME occurrences^4–8,16^. All of these magnetic activity structures influence the conditions in the interplanetary magnetic field (IMF) and thus geomagnetic field through interactions of the IMF and solar wind plasma. The solar wind is a stream of solar plasma carrying the frozen-in IMF throughout the interplanetary medium^26^. Interactions of the IMF and solar wind plasma with the geomagnetic field results in a dynamic terrestrial magnetosphere. Although Earth’s magnetosphere creates a cavity in the solar wind, mass, momentum and energy continuously transfer to Earth’s magnetosphere-ionospheric system with the amount of energy transferred controlled by the direction and strength of the IMF. Geomagnetic field measurements at geostationary orbit are useful for both operational and scientific purposes because this location is effective for monitoring major magnetospheric current systems^29^.

In summary, using multi-GOES mission data for the past 3 solar cycles, we for the first time show variations in Earth’s magnetic field with periods that can be associated with the solar QBOs. These fluctuations occur in the interior of the Sun and propagated to Earth’s geospace and throughout the Heliosphere via the solar wind, source regions of which are CHs.

## Methods

### Earth’s magnetic field data from multi-GOES missions

Magnetic field measurements at geostationary orbit by GOES satellites started with GOES-01 mission in 1977 and has been continuing since then through follow-on GOES missions^29,35,36^. In our study, we used the 1-min average magnetic field data from NCEI-NOAA servers (https://www.ncei.noaa.gov/) for missions GOES-01 to GOES15, spanning the time period from the 1st of August 1977 to the 2nd of March 2020. Additionally, we used L2 high-resolution data from the same server extending from the 1st of July 1995 (GOES-08) to the 3rd of July 2023 (GOES-16).

The magnetic field measurements are most commonly available in the spacecraft EPN coordinate system. In the EPN system, the E-axis is the Earthward (nadir), the P-axis is the normal to the orbital plane (Poleward), and the N-axis (Normal) completes the right-handed system^37^. We then converted the magnetic field data from the EPN frame to the GSM (results shown in Fig. 1). The GSM frame has its origin at the center of Earth and x-axis defined along the Earth-Sun line positive towards the Sun. The y-axis is the cross product of the x-axis and the Earth’s magnetic dipole axis, defined as the azimuthal component that is positive towards dusk magnetic local time. The z-axis is defined as the cross product of the x and y components of the GSM. There were some days where the conversion could not be applied because of unavailability of orbital two-line element sets of the satellites.

Following the conversion of the entirety of data extending from 1976 to 2023, we removed the possible outliers using 4$\sigma$ standard deviation around the mean as a threshold value, above (below) which is identified as an outlier. The $\sigma$ standard deviation value is calculated for each GOES mission data.

### Pre-processing the magnetic field data of the Earth and Sunspot Numbers

We first binned data for each GOES mission, separately, using 3-month-bins. One criterion we used is the fullness of the 3-month bins. For example, if the 3-month bins are not 75% full, then this bin is considered as empty and not included in the further analyses. We then calculated the mean and standard deviation of the magnetic field in each full bin for each GOES mission. This method, naturally, resulted in data gaps in magnetic field measurements. To avoid any undesirable effects to the results from any interpolation method, as a second data selection criterion, we chose periods where the GOES magnetic field data has its maximum continuous extend, which ranged from 1.5 to 8.2 years. Since the scope of our work is the QBOs, the period of which is in the order of 1.3–1.6 years, as the third data selection criterion, we disregarded data the length of which is shorter than 4.5 years.

In the end, there were only 8 GOES missions that satisfied our 3 data selection criteria, which are GOES-06, GOES-07, GOES-08, GOES-10, GOES-12, GOES-13, GOES-15 and GOES-17 (the left panels of Fig. 2).

Further, we band-pass filtered the data using a Butterworth filter of order 5 for the frequency band between (1/4.5) and (1/1.1) year$^{-1}$ as the longer-term variations in the data could suppress the QBO signals (the right panels of Fig. 2). These cut-off frequencies are chosen to remove the orbital effects that might be inherently in the data as well as the known dominant effects of the Schwabe cycles on the QBOs and its potential 2nd harmonics. The Butterworth filter, which is maximally flat in the pass-band and does not cause any distortion in the low-frequency signal component^38^, is defined as;

1 $$\begin{aligned} \mid H_{B}(j\omega )\mid ^{2} = \frac{1}{1+(\omega / \omega _{c})^{2n}},\end{aligned}$$

where $\omega _{c}$ and *n* denote the cut-off frequency and the order of the Butterworth filter. To filter the data we used *SciPy package*^39^ in python, which allows to digitally filter the data forwards and backwards to achieve zero phase filtering of the data.

All of the above steps, which were used to pre-process the geomagnetic field data obtained from the GOES missions are also applied to the monthly sunspot numbers obtained from WDC-SILSO, Royal Observatory of Belgium, Brussels^30^.

### Wavelet analyses

To detect the periodicities in the data sets, we first calculated their continuous wavelet transformation (CWT) spectra using *pycwt* library in python. Prior to the analyses, the underlying data is standardized by their individual mean and standard deviation values.

The CWT are used to detect the dominant modes of variability and their temporal evaluations in time series data by decomposing them^40^. A wavelet is defined as a function with zero mean which is localized in both frequency (or period) and time domains^41^. Among different wavelet functions, the most commonly used wavelet function is the Morlet wavelet that is a complex symmetric function and is defined as

2 $$\begin{aligned} \psi _{0} (\eta ) = \pi ^{-1/4} e^{i \omega _{0} \eta } e^{-\eta ^{2}/2} \end{aligned}$$

where $\omega _{0}$ and $\eta$ are the dimensionless frequency and time^40^. The choice of $\omega _{0} = 6$ provides a good balance between time and frequency localisation that can also detect peaks and valleys^41,42^. The CWT of a time series that has uniform time steps of $\delta t$ is defined as the convolution of time series ($x_n$) with the scaled and normalized wavelet and is given by

3 $$\begin{aligned} W^{X}_{n} (s) = \sqrt{\delta t/s}\sum _{n' = 1}^{N} x_{n'}\psi _{0} \left[ \left( n' - n \right) \delta t/s \right] \end{aligned}$$

where *s* is scale. The wavelet power is then calculated as $|W^{X}_{n} (s)|^2$, where the complex argument of $W^{X}_{n} (s)$ is the local phase^40,41^.

The statistical significances of the CWT are calculated using Monte Carlo method based on the each time series first order auto-regressive correlation coefficients (AR1). In our study, we used 500 ensembles of surrogate data set pairs with the same AR1 coefficients as the input data sets.

Together with their CWTs, we also calculated their global wavelet spectra, which is calculated by averaging over all the local wavelet spectra in time and is given as^40^;

4 $$\begin{aligned} {\overline{W}}^{2}_{n} (s) = \frac{1}{N}\sum _{n = 0}^{N-1}\mid W_{n} (s) \mid ^{2} \end{aligned}$$

Global wavelet (GW) spectrum provides a measure for the background variation in the data as well as their consistent and unbiased estimation of the true power spectrum^40^. The average frequency resolution for all the GW spectra is calculated to be (1/0.83) year$^{-1}$ for every data set.

## Data Availability

The data sets used and/or analysed during the current study are available from the corresponding author on reasonable request.
